# Association of Laparoscopic Surgery with Improved Perioperative and Survival Outcomes in Patients with Resectable Intrahepatic Cholangiocarcinoma: A Systematic Review and Meta-Analysis from Propensity-Score Matched Studies

**DOI:** 10.1245/s10434-023-13498-0

**Published:** 2023-04-28

**Authors:** Daniel Aliseda, Gonzalo Sapisochin, Pablo Martí-Cruchaga, Gabriel Zozaya, Nuria Blanco, Brian K. P. Goh, Fernando Rotellar

**Affiliations:** 1 HPB and Liver Transplant Unit. Department of General Surgery, Clinica Universidad de Navarra, University of Navarra, Pamplona, Spain; 2 Institute of Health Research of Navarra (IdisNA), Pamplona, Spain; 3 Abdominal Transplant and HPB Surgical Oncology, Division of General Surgery, Ajmera Transplant Center, Toronto General Hospital, University of Toronto, Toronto, ON Canada; 4 Department of Hepatopancreatobiliary and Transplant Surgery, Singapore General Hospital and National Cancer Centre, Singapore, Singapore; 5 Duke-National University Singapore Medical School, Singapore, Singapore

## Abstract

**Background:**

Recent studies have associated laparoscopic surgery with better overall survival (OS) in patients with hepatocellular carcinoma (HCC) and colorectal liver metastasis (CRLM). The potential benefits of laparoscopic liver resection (LLR) over open liver resection (OLR) have not been demonstrated in patients with intrahepatic cholangiocarcinoma (iCC).

**Methods:**

A systematic review of the PubMed, EMBASE, and Web of Science databases was performed to search studies comparing OS and perioperative outcome for patients with resectable iCC. Propensity-score matched (PSM) studies published from database inception to May 1, 2022 were eligible. A frequentist, patient-level, one-stage meta-analysis was performed to analyze the differences in OS between LLR and OLR. Second, intraoperative, postoperative, and oncological outcomes were compared between the two approaches by using a random-effects DerSimonian-Laird model.

**Results:**

Six PSM studies involving data from 1.042 patients (530 OLR vs. 512 LLR) were included. LLR in patients with resectable iCC was found to significantly decrease the hazard of death (stratified hazard ratio [HR]: 0.795 [95% confidence interval [CI]: 0.638–0.992]) compared with OLR. Moreover, LLR appears to be significantly associated with a decrease in intraoperative bleeding (− 161.47 ml [95% CI − 237.26 to − 85.69 ml]) and transfusion (OR = 0.41 [95% CI 0.26–0.69]), as well as with a shorter hospital stay (− 3.16 days [95% CI − 4.98 to − 1.34]) and a lower rate of major (Clavien-Dindo ≥III) complications (OR = 0.60 [95% CI 0.39–0.93]).

**Conclusions:**

This large meta-analysis of PSM studies shows that LLR in patients with resectable iCC is associated with improved perioperative outcomes and, being conservative, yields similar OS outcomes compared with OLR.

**Supplementary Information:**

The online version contains supplementary material available at 10.1245/s10434-023-13498-0.

Cholangiocarcinoma (CC) is a rare cancer; however, its incidence and mortality have been increasing worldwide over the past few decades, and it is currently the second most common primary hepatic tumor.^1^ CC is usually asymptomatic during the early stages. Hence, most patients present with metastatic or locally advanced disease. Therefore, less than 25% of patients are candidates for surgery at diagnosis.^2^ Intrahepatic cholangiocarcinoma (iCC) accounts for approximately 10–20% of all patients and usually presents as large tumors.^1^ Consequently, patients with initial resectability usually require  a major hepatectomy to achieve R0 resection.^3^ Because approximately 40% of patients present with lymph node involvement, it is recommended to perform a portal lymphadenectomy retrieving at least six lymph nodes to achieve adequate staging.^4^ Despite adequate oncological resection, patients with iCC experience high recurrence rates and have a modest prognosis; 5-year overall survival (OS) is approximately 25–40%.^5,6^ This OS has remained stable over the past decade, with adjuvant capecitabine therapy providing a modest benefit in disease-free survival (DFS) but not OS in the intention-to-treat analysis.^7^

For primary and metastatic tumors, laparoscopic liver resection (LLR) has been shown to be advantageous over open liver resection (OLR) in terms of intra- and postoperative outcomes.^8,9^ Moreover, it has been postulated that a decrease in blood loss, transfusion rate, and morbidity associated with LLR may have a positive impact on OS.^10–13^ Indeed, high-quality meta-analyses have recently found superior OS in patients with laparoscopically resected colorectal liver metastases (CRLM) and in cirrhotic patients with hepatocellular carcinoma (HCC).^14,15^ However, the benefits of the laparoscopic approach in patients with iCC remain uncertain. The purpose of this systematic review and meta-analysis was to assess the possible beneficial effects of laparoscopic liver surgery compared to open surgery in patients with iCC.

## Material and Methods

A systematic review was conducted according to the PRISMA (PRISMA) guidelines, following Cochrane recommendations, and registered *a priori* in PROSPERO (CRD42022330665).^16,17^

### Search Strategy and Study Selection

Three electronic databases (PubMed, Embase, and Web of Science) were searched by using Medical Subject Headings (MeSH) and keywords to retrieve studies published in English from database inception to May 1, 2022. All eligible studies published in peer-reviewed journals comparing postoperative and survival outcomes between OLR and LLR in patients with resectable iCC were considered. Randomized controlled trials and propensity-score matched studies (PSM) reporting any type of liver resection (LR) were included. Studies that included liver transplantation or liver surgery for other tumor types were excluded, as were studies reporting patients treated with robotic, hybrid, or hand-assisted approaches. For survival analysis, only studies that reported Kaplan–Meier curves describing the OS of the entire cohort were included.

The following key terms were used to identify relevant studies: “liver OR hepatic” AND “intrahepatic cholangiocarcinoma OR cholangiocarcinoma OR ICC” AND “surgery OR resection OR hepatectomy” AND “laparoscopic OR open.” All possible combinations of keywords were used, and an additional cross-reference search was performed. After removing duplicate articles, two reviewers (DA and NB) independently screened the titles and abstracts by performing the first double-blind selection. Subsequent identification of the articles to be included was performed in duplicate (DA and NB) by reading the full texts. Rejected articles were correctly identified, and noncompliance with the inclusion criteria is indicated in eTable 1. Discrepancies at every step were resolved through consensus and were achieved for all included studies. Two authors (DA and NB) independently extracted data using a customized form created specifically for this study. Information about baseline patient and tumor characteristics, preoperative analyses, operative details, and long-term survival was collected.

### Objective of the Study

The primary endpoint was to identify differences in the OS measured in months after LR. The secondary outcomes were differences in intra-, postoperative, and oncological outcomes, defined as follows:

1. Intraoperative: duration (minutes), blood loss (ml), and blood transfusion (number).
2. Postoperative: length of hospital stay (days), overall morbidity (according to Clavien-Dindo^18^), major complications (Clavien-Dindo ≥ III), and perioperative mortality (up to 90 days).
3. Oncological: R0 resection and lymph node retrieval (number).

### Assessment of the Quality of Evidence

The methodological quality of the selected studies was assessed using the Newcastle-Ottawa Scale (NOS).^19^ Eight items were assessed in three key domains: selection, comparability, and outcome. The quality of the studies was categorized into three levels according to the number of points obtained: low (<4 points), moderate (between 4 and 6 points), and high (≥7 points). The evaluation was conducted in duplicate and independently by two reviewers (DA and NB). Disagreements were resolved through consensus.

### Statistical Analysis

#### Reconstruction of Time-to-Event Outcomes

Survival data were extracted from Kaplan–Meier curves using the Digitizelt software. Patient-level survival data were used to estimate time-to-event outcomes by using an iterative algorithm based on the Kaplan–Meier estimation method proposed by Guyot et al.^20–22^ To correct the values for violators to ensure monotonicity, the pool-adjacent-violators algorithm was used to ensure the monotonicity constraint.^22^ Before analysis, the reconstructed Kaplan–Meier data were examined by checking the original published plots, 1- to 5- year OS rates, log-rank values, and number-at-risk tables.

#### Survival Analysis

A Cox proportional hazards model was used to calculate hazard ratios (HR). A stratified estimation was performed to fit separate Cox proportional hazards models, assuming equal coefficients but different baseline hazard functions, and was conducted as the primary analysis. Second, to model within-group correlation, a shared-frailty model was used. The marginal Cox proportional hazards model also was conducted. The Grambsch-Therneau test, the plotted scaled Schoenfeld residuals, and predicted versus observed survival functions were used to identify violations of the proportionality assumption of Cox regression models.^23^ Between-group contrast measures calculated from the restricted mean survival time (RMST) were performed using the Naïve Kaplan-Meier method as an alternative to the hazard ratio.^24^ Second, we performed a two-step meta-analysis of aggregated HR (calculated independently from each study) using a fixed-effects model (inverse variance). The Kaplan-Meier product-limit model was used to estimate time-to-event outcomes, and the log-rank test was used to compare unadjusted OS.

#### Metanalysis of Aggregated Patient Data

Analyses were performed by using odds ratios (OR) with 95% confidence intervals (CI) for dichotomous variables, and weighted mean differences with 95% CI for continuous variables. In cases where studies reported only the median, range (or interquartile range), and sample size, the formulas proposed by Luo et al. and Wan et al.^25,26^ were used to calculate mean values and standard deviation, respectively. A random effects DerSimonian and Laird model was used to meta-analyze the data. Heterogeneity was evaluated using the Cochrane Q test and *I*^2^. The Higgins statistic (*I*^2^) was used to quantify the proportion of total variability across studies resulting from heterogeneity rather than chance. *I*^2^ values of 25%, 50%, and 75% were defined as low, moderate, and high heterogeneity, respectively.^27^ Publication bias was tested using the “metafunnel” and “metabias” functions in STATA, explored visually using funnel plots, assessed quantitatively using Egger’s test, and was considered to exist when *p* < 0.10.

The meta-analysis was conducted using STATA version 16 (StataCorp, College Station, TX). All tests were two-sided, with a significance level of 0.05.

## Results

### Systematic Search

**Fig. 1:**
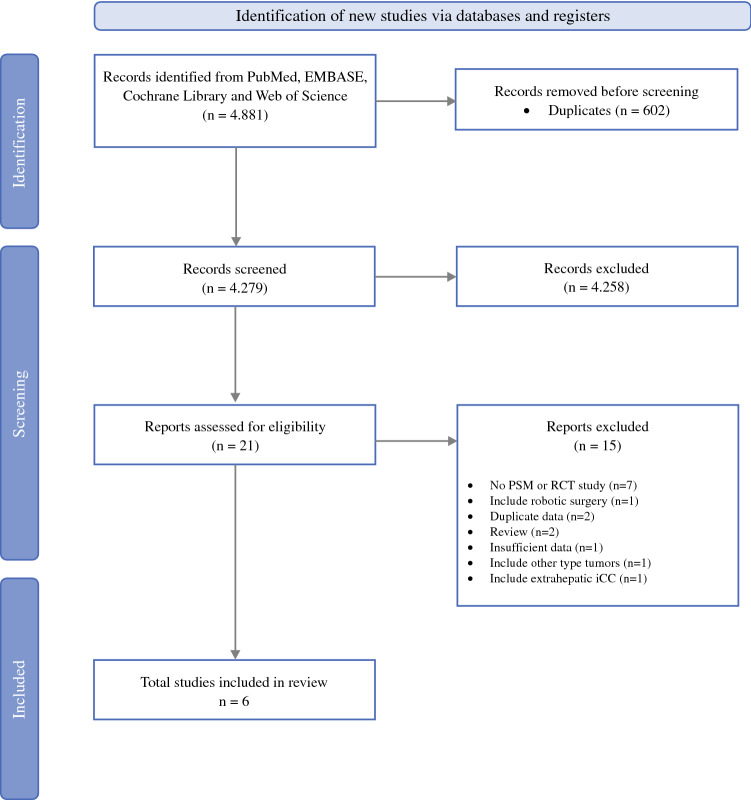
PRISMA flowchart according to guidelines

The search yielded 4.881 potentially relevant articles. After the removal of duplicates and the first screening of abstracts, 21 articles were analyzed in detail by reading the full text (Fig. 1). Finally, six articles met all eligibility criteria. The systematic search strategy, the articles finally not selected, and the reasons for rejection are available in eTable 1.

### Study and Patients Baseline Characteristics

Six PSM studies^28–32^ and no RCT involving 1.042 patients were included. The studies were conducted in both Eastern (China and South Korea) and Western (Italy and France) centers. Among them, 530 underwent OLR and the remaining 512 underwent LLR. The characteristics of the six studies as well as patient, tumor, and operative details, are shown in Table 1.

**Table 1:** Characteristics of studies

Study (yr)	Study Period	PSM adjusted for	No. patients	Group	Patients characteristics	Tumor characteristics	TNM stage	Preoperative biochemical parameters	Operative details
Jinhuan et al.^31^ (2022)	January 2011-December 2018	Sex, age, BMI, smoking drinking status, tumor size, tumor number, TNM stage, differentiation, lymphatic invasion, vascular invasion, nerve invasion, HBV infection, hepatolithiasis, diabetes, cirrhosis, previous abdominal surgery, Child–Pugh classification, resection range, Charlson Comorbidity Index score, and anatomical resection	122	Open	73 men, 49 women Age > 65 yr: 33 (27.0%); ASA III: 3 (2.46%); cirrhosis: 27 (22.1%), PHT 2 (1.64%)	84.4% solitary tumors. Median size 5 cm (IQR 3,5-6 cm); 91.8% moderately or poorly differentiated; 5.74% lymphatic invasion	I: 65 (53.3%) II: 25 (20.5%) III: 32 (26.2%)	Median platelet count: 220 x 109/L (IQR 173–256) Mean AST: 24.5 (IQR 20–33.8) Mean ALT: 21.0 (IQR 14.0–33.0) Median albumin: 39.9 g/L (IQR 37.6–44.2) Median bilirubin: 10.9 (IQR 7.40–14.5) Median PT (s) 12.6 (IQR 11.9–13.5) Median CEA 3.12 ng/mL (1.81–9,41) Median Ca 19.9 (kU/L) 67.0 (IQR 19.0–557]	49.2% Major hepatectomies 38.5% Anatomic resections
			122	Laparoscopic	68 men, 54 women Age > 65 yr: 43 (35.2%); ASA III: 14 (11.5%); Cirrhosis: 28 (23.0%), PHT 2 (1.64%)	82.8% solitary tumors. Median size 4.35 cm (IQR 3–6 cm); 88.5% moderately or poorly differentiated; 4.10% lymphatic invasion	I: 66 (54.1%) II: 25 (20.5%) III: 31 (25.4%)	Median platelet count: 214 x 109/L (IQR 178–288) Mean AST: 24.0 (IQR 20–34.0) Mean ALT: 21.0 (IQR 14.2–40.8) Median albumin: 42.2 g/L (IQR 39.3–45.3) Median bilirubin: 12.6 (IQR 9.16–16.6) Median PT (s) 12.8 (IQR 11.8–13.6) Median CEA 2.84 ng/mL (1.59–5.00) Median Ca 19.9 (kU/L) 46.2 (IQR 16.7–405]	45.9% Major hepatectomies 50.8% Anatomic resections
Ratti et al.^29^ (2021)	LLR between 2014 and 2020 OLR between 2004 and 2020	Age, ASA score, presence of chronic liver disease, Ca 19.9 serum level, tumor dimension, number of lesions and tumor stage	150	Open	86 men, 64 women Mean age 62 yr ±7 ASA III: 36 (24%); Cirrhosis: 13 (8.7%)	70% solitary tumors. Mean size 5.8 cm ± 1.2; 92% moderately or poorly differentiated; 35.3% lymphatic invasion	I/II: 93 (62%) III/IVa: 57 (38%)	Mean CEA 35 ng/mL ± 19 Mean Ca 19.9 U/mL 93 ± 64	36.7% Major hepatectomies 90% Associated lymphadenectomy
			150	Laparoscopic	92 men, 58 women Mean age 61 yr ± 4 ASA III: 38 (25.3%); Cirrhosis: 20 (13.3%)	72% solitary tumors. Mean size 5.3 cm ± 2,3; 88% moderately or poorly differentiated; 37.3% lymphatic invasion	I/II: 91 (60.7%) III/IVa: 59 (39.3%)	Mean CEA 22 ng/mL ± 21 Mean Ca 19.9 U/mL 89 ± 76	34% Major hepatectomies 88% Associated lymphadenectomy
Brustia et al.^30^ (2021)	January 2000 up to June 2018	Difficulty classification (Institut Mutualiste Montsouris), patient’s age (<50 years, 50–70 yr, > 70 yr), year of surgery (2000–2018), number of lesions, and tumor size (max. diameter).	89	Open	38 men, 51 women Mean age 67.92 yr ± 8.97 ASA III 24 (40.0%) F3-F4: 6 (8.0%)	84.3% solitary tumors, Mean size 5.32 cm ± 3.73	NR	NR	69.7% Major resection 21.3% Associated lymphadenectomy
			89	Laparoscopic	52 men, 37 women Mean age 65.24 yr ± 11,40 ASA III: 28 (38.4%) F3-F4: 21 (24.7%)	86.5% solitary tumors, Mean size 4.67 cm ± 2.56	NR	NR	52.8% Major resection 17.9% Associated lymphadenectomy
Hobeika et al.^33^ (2021)	January 2000 and November 2017	ASA grade III or more, tumor size ≥5 cm, portal vein embolization, extent of resection and liver resection difficulty level	109	Open	Median age 61 yr (IQR 52–68) Severe fibrosis (F3-F4): 21 (19.3%)	Tumor size > 5 cm: 45 (41.3%) 38% lymphatic invasion	NR	NR	47.7% Major hepatectomies 25.7% Lymphadenectomy*
			109	Laparoscopic	Median age 67 yr (IQR 60–72) Severe fibrosis (F3-F4): 30 (27.5%)	Tumor size > 5 cm: 45 (41.3%) 19% lymphatic invasion	NR	NR	47.7% Major hepatectomies 14.7% Lymphadenectomy*
Kang et al.^28^ (2020)	From August 2004 to October 2015	Age, gender, tumor location (unfavorable = S7, S8; favorable = otherwise), extent of hepatectomy (major resection or minor resection), and nodularity (single or multiple)	24	Open	15 men, 9 women Mean age 68.1 yr ± 10.2 12.5% Cirrhosis	91,7% solitary tumors, Mean size 4.1 cm ± 1.8 100% Adyuvant therapy (CT or CRT)	NR	NR	75% Major resection 70.8% Associated lymphadenectomy
			24	Laparoscopic	15 men, 9 women Mean age 66.8 yr ± 9.7 12.5% Cirrhosis	91,7% solitary tumors, Mean size 4.7 cm ± 3.3 100% Adyuvant therapy (CT or CRT)	NR	NR	75% Major resection 25% Associated lymphadenectomy
Zhu et al.^32^ (2019)	January 2012 to June 2017	Age, tumor characteristics (tumor size, tumor number, nodule status, tumor differentiation, and microvascular invasion), ASA grade, underlying liver cirrhosis, liver function (Child Pugh grade), and resection extent	36	Open	19 men, 17 women Mean age 55.6 yr ± 9.8 ASA II: 4 (11.1%); Cirrhosis: 15 (41.7%)	77.8% solitary tumors, Median size 6 cm (range 4–9) 75% poorly differentiated 25% lymphatic invasion	NR	Mean ALT (IU/L) 33.8 ± 22.9 Mean Bilirrubin (umol/L) 14.0 ± 6.9 Mean platelet count (10^9^/L) 159.0 ± 75.0 Mean PT (s) 11.5 ± 0.8 Mean albumin (g/L) 43.1 ± 3.2 Median Ca 19.9 U/mL 31 (range 0.6–1000)	61.1% Major resection^+^ 41.7% Associated lymphadenectomy
			18	Laparoscopic	10 men, 8 women Mean age 54.1 yr ± 16.6 ASA II: 2 (11.1%); Cirrhosis: 6 (33.3%)	77.8% solitary tumors, Median size 6 cm (range 3–9) 66.7% poorly differentiated 16.7% lymphatic invasion	NR	Mean ALT (IU/L) 33.8 ± 23.5 Mean bilirubin (umol/L) 13.6 ± 9.5 Mean platelet count (10^9^/L 165.3 ± 74.3 Mean PT (s) 11.8 ± 1.3 Mean albumin (g/L) 42.8 ± 4.8 Median Ca 19.9 U/mL 60.8 (range 0.6–1000)	55.6% Major resection^+^ 38.9% Associated lymphadenectomy

*PSM* propensity-score matched; *BMI* body mass index; *ASA* American Society of Anesthesiologists; *PHT* portal hypertension; *CT* chemotherapy; *CRT* chemoradiotherapy; *PT* protombin time; *NR* not reported

*Lymphadenectomy according to AJCC (8th version): at least six nodes harvested; + Right posterior seccionectomy considered as major resection. In the remaining studies, major resection was considered when > 3 liver segments were resected

### Study Quality Assessment

The assessment of the quality of the studies and the scores in each of the eight domains of the NOS scale are specified in eTable 2. In summary, all studies obtained an NOS score of ≥7 stars, indicating high methodological quality, except for one study that was considered to be of moderate quality.

### Survival Analysis

Five studies^28–32^, comprising 824 patients (421 in the OLR group and 403 in the LLR group), met the inclusion criteria for survival analysis. The survival data reconstruction yielded similar patient-level survival data compared with the original plots, and all the included studies complied with the proportional hazard assumption (Table 2 and Online supplementary material). In stratified analysis, we found a significant difference in OS depending on the surgical approach adopted. Laparoscopic liver resection in patients with resectable iCC was significantly associated with a reduced hazard of death (stratified hazard ratio [HR] = 0.795 [95% confidence interval [CI]: 0.638–0.992; *P =* 0.041]) compared with the open approach. This significant difference in OS was more pronounced in the marginal model (HR = 0.768 [95% CI 0.617–0.957; *P =* 0.018]) and the shared frailty model (HR = 0.780 [95% CI 0.626–0.972; *P =* 0.028]).

**Table 2:** Original and reconstructed curves from the included studies

Author	Original curves	Reconstructed curves
Jinhuan et al.^31^		
Brustia et al.^30^		
Ratti et al.^29^		
Kang et al.^28^		
Zhu et al.^32^		

**Fig. 2:**
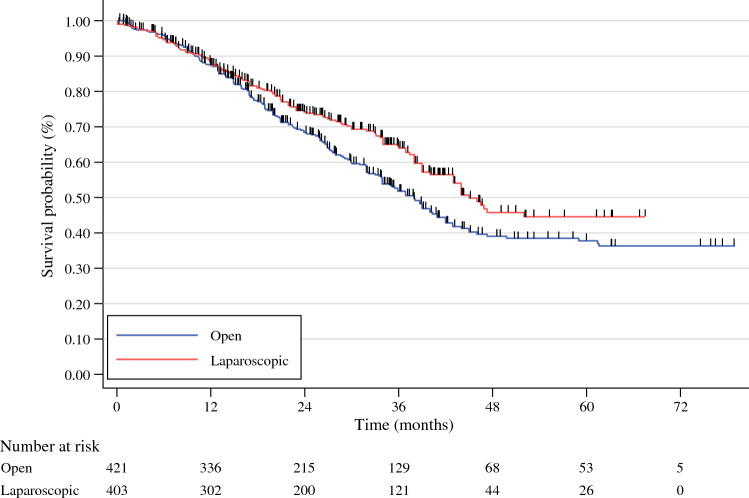
Kaplan-Meier curves and number-at-risk table for 824 patients (log-rank *P* = 0.041)

Based on the reconstructed patient-level survival data, the RMST was 3.6 months (*P* = 0.027) higher among patients undergoing LLR, which corresponds to an increase in the relative life expectancy of 7.7% at 5 years. The OS rates at 1, 3, and 5 years were 87.4% [95% CI 83.6–90.4], 64.0% [95% CI 58.0–69.3], and 44.6% [95% CI 36.7–52.1], respectively, for patients who underwent laparoscopic surgery and 87.4% [95% CI 83.7–90.2], 51.8% [95% CI 46.2–57.2] and 37.8% [95% CI 31.7–43.8], respectively, for patients who underwent open surgery (log-rank *P* = 0.041; Fig. 2). Using the inverse variance weighting model for the two-step meta-analysis, the pooled HR was 0.80 (95% CI 0.84–0.99; *P* = 0.04) (*I*^2^ = 0.00%; *P* = 0.67) (eFig. 10). A summary of all the survival analyses conducted is presented in Table 3.

**Table 3:** Primary and sensitivity analyses of OS estimates using reconstructed survival information

	Relative effect (95% CI)	*P* value	Test of non-PH
Semiparametric models			
Stratified HR	0.795 (0.638–0.992)	0.041	0.5791
Marginal HR	0.768 (0.617–0.957)	0.018	0.7449
Shared frailty model HR	0.780 (0.626–0.972)	0.028	0.7095
Nonparametric models			
RMST difference (up to 5 yr)	+3.62 months (0.41–6.83)	0.027	
RMST ratio (up to 5 yr)	1.096 (1.01–1.18)	0.027	

*HR* hazard ratio; *RMST* restricted median survival time; *PH* proportional hazard

### Surgical Outcomes

Five studies^28,29,31–33^ involving 864 patients (441 in the OLR group and 423 in the LLR group) reported differences in the intraoperative parameters and postoperative outcomes. Data on operative duration were reported in five studies^28,29,31–33^ with a higher significant mean difference (MD) of 24.68 minutes (95% CI 2.07–47.28 min; *P* = 0.032) in the LLR group with a moderate degree of heterogeneity between studies (*I*^2^ = 69.2%; *P* = 0.01; eFig. 1). Overall, four studies reported intraoperative blood loss.^28,29,32,33^ LLR in patients with iCC was associated with a significantly lower blood loss (MD: − 161.47 ml [95% CI − 237.26 to − 85.69 ml; *P* = 0.0001]) (*I*^2^ = 51.2%; *P* = 0.10; eFig. 2). The intraoperative transfusion rate was provided in four studies.^29,31–33^ Laparoscopic resection was associated with a significantly reduced risk of transfusion (OR = 0.42 [95% CI 0.26–0.69; *P* = 0.0006]) (*I*^2^ = 0.00%; *P* = 0.79; eFig. 3).

Pooled data from five studies reported that laparoscopic surgery was associated with a significant decrease of 3.16 days of hospital stay (95% CI − 4.98 to − 1.34 days; *P =* 0.0007) (*I*^2^= 92.24%; *P* ≤ 0.0001; eFig. 4).^28,29,31–33^ Four studies reported that the risk of perioperative mortality was increased in patients operated on using a laparoscopic approach, although this difference was not statistically significant (OR = 1.23 [95% CI 0.53 to 2.85;* P =* 0.63]) (*I*^2^ = 0.00%; *P* = 0.96; eFig. 5).^29,31–33^ A significant association was observed for major complications (Clavien-Dindo ≥ 3). Four studies reported that LLR was significantly associated with a 40% reduced risk of major complications (OR = 0.60 [95% CI 0.39–0.93; *P* = 0.023]) (*I*^2^ = 0.00%; *P* = 0.49; eFig. 7).^29,31–33^ In addition, LLR was associated with a lower rate of overall complications, although this was not statistically significant (OR = 0.80 [95% CI 0.45–1.42; *P=* 0.44]) (*I*^2^ = 47.98%; *P* = 0.12; eFig. 6). Pooled data from three studies showed non-significant differences between surgical approaches in relation to the risk of achieving oncologic surgery (R0) (OR = 1.10 [95% CI 0.58–2.10; *P =* 0.762]) (*I*^2^ = 0.00%; *P* = 0.68; eFig. 8).^29,32,33^ According to four studies, no significant difference in the performance of lymphadenectomy was observed between both groups (OR = 0.52 [95% CI 0.27–1.01; *P =* 0.054]) (*I*^2^ = 53.3%; *P* = 0.09; eFig. 9A).^28,29,32,33^ Information on lymph node retrieval was reported in two studies.^29,33^ Open liver resection was associated with a significantly lower number of retrieved lymph nodes (MD − 1.69 nodes [95% CI − 1.99 to − 1.39; *P* = 0.001]) (*I*^2^ = 0.00%; *P* = 0.90; eFig. 9B).

## Discussion

This meta-analysis of PSM studies showed that LLR was significantly associated with improved overall survival compared to OLR in patients with resectable iCC. Laparoscopic resection resulted in a 20.5% reduced hazard of death compared with the open approach. This association in favor of laparoscopy was consistent across different analyses. The results of this meta-analysis show benefits not only in terms of survival but also in terms of intraoperative parameters and postoperative outcomes, with an association of LLR with reduced intraoperative bleeding, need for intraoperative blood transfusion, shorter hospital stay, and lower rates of major complications. These findings are both promising and provocative, because liver surgery for iCC is extremely demanding. The existing data in the literature still raise concerns about the advantages of laparoscopy in these patients. The concrete reasons why laparoscopy may improve the OS of patients with iCC are still not known with certainty, although it is likely a combination of correct patient selection, intra- and postoperative benefits as well as immunobiological factors.^34–36^

The potential survival benefit of the laparoscopic approach over the open approach has previously been postulated in the field of liver surgery. Recent, high-quality meta-analyses have associated LLR with improved OS in patients with CRLM and cirrhotic patients with HCC.^14,15^ This association also has been demonstrated in other surgical procedures, such as rectal surgery.^37^ In this scenario, the survival advantage takes on a particularly important value, as the prognosis of patients with resectable iCC has remained stable over the past decade. Our study suggests a possible advantage of the laparoscopic approach in patients with resectable iCC.

Tumor-promoting inflammation and evasion of the immune system are considered to be the main biological capabilities during the development of human tumors.^38^ Immune function and inflammatory processes differ between patients undergoing laparoscopic and open surgery. Clinical and experimental studies shown that LLR reduces the secretion of proinflammatory factors, such as IL-6, C-reactive protein, TNF alpha, or NFkB and preserves better postoperative immunity.^35,39,40^ These two factors appear to play an important role in tumor development and metastasis as well as in the production of tumor angiogenesis and secretion of tumor-promoting mitogens, which could lead to cancer recurrence and negatively impact survival.^41^

Furthermore, intraoperative bleeding associated with perioperative transfusion has both postoperative and long-term impact.^36^ In particular, several studies on liver surgery in iCC patients have shown an association between increased transfusion and higher rates of overall and major complications, as well as a lengthening of hospital stay.^42^ Regarding survival, transfusion is an independent factor for worse OS and DFS in patients undergoing resection for distal cholangiocarcinoma, as well as some secondary liver tumors, such as CRLM.^13,43^ In this setting, LLR has consistently demonstrated a reduction in intraoperative bleeding compared with the open approach for primary and metastatic tumors.^8,9^ As this study shows, patients who underwent resection for iCC also appear to have a lower risk of transfusion, which is probably a contributing factor to the improvement of postoperative complications and also may have a positive impact on OS.

The pursuit of strategies that decrease the incidence of complications is of vital concern, because they increase the time to initiation of adjuvant therapy, have a physical and emotional impact on the patient, and increase the economic cost. As with other tumors, complications after LR for iCC have been shown to worsen survival. In fact, Spolverato et al. demonstrated that both morbidity and severity of complications affect OS and increase the risk of disease recurrence and long-term disease-specific death by > 50%.^34^ In this setting, LLR has been shown to decrease overall and major morbidity in patients undergoing surgery for HCC and CRLM.^11,14^ For patients resected for iCC, although this has not been proven, no evidence suggests that this benefit could be different from that of other tumors. In our study, we have shown that laparoscopic surgery is significantly associated with a lower risk of major complications compared with OLR.

Although controversial, hilar lymphadenectomy should be performed to help in staging and guiding adjuvant treatment, and appears to be associated with prolonged OS in node-negative patients.^44,45^ Some authors postulated that the laparoscopic approach poses a risk in performing correct lymphadenectomy. However, in the present study, LLR was significantly associated with a higher number of retrieved LNs and did not result in increased morbidity. However, this result is most likely affected by the increase in the number of lymphadenectomies performed and lymph nodes harvested in recent years, and should be cautiously interpreted.^46^

In addition to the reasons mentioned earlier, and despite the inclusion of PSM studies, one key aspect undoubtedly has an impact on the results of this study and should be highlighted. This is the careful selection of patients for LLR. Laparoscopic liver surgery for iCC is technically challenging. Therefore, a number of factors related to the experience of the surgical team, the patient, the tumor, and the type of LR to be performed influence the choice of open versus laparoscopic approach. Thus, proper patient selection is likely to be just as important, if not more important, than the technique itself, as it is the first step toward achieving optimal perioperative and survival outcomes.

Despite the inclusion of only PSM studies, the results presented are not completely protected against selection bias. The quality of the propensity score model, choice of matching algorithm, and availability and completeness of data can all affect the potential for selection bias in PSM studies. Additionally, there may be unmeasured confounding variables that could affect the estimation of the treatment effect. Therefore, the results presented should be interpreted with caution. Furthermore, the era effect, presence of heterogeneity in some analyses and publication bias need to be considered. Future studies should be conducted to determine the role of laparoscopy in inflammatory, immune, and other processes that, together with improved perioperative outcomes, may explain why LLR appears to confer a survival benefit in liver surgery.

## Conclusions

This large meta-analysis of patient-level survival data provides evidence to support laparoscopic surgery in patients with iCC. LLR was associated with a significantly longer OS than open surgery in all survival analyses performed. On a conservative basis, this suggests that in well-selected patients, laparoscopic surgery offers OS outcomes at least equivalent to those of OLR. LLR also was associated with less intraoperative bleeding and transfusion, shorter hospital stay, and lower rate of major complications.

## Supplementary Information

Below is the link to the electronic supplementary material.

Supplementary file1 (DOCX 4465 kb)
