# Supplementary material for: Association of Laparoscopic Surgery with Improved Perioperative and Survival Outcomes in Patients with Resectable Intrahepatic Cholangiocarcinoma: A Systematic Review and Meta-Analysis from Propensity-Score Matched Studies
Source: Ann Surg Oncol. 2023 Apr 28;30(8):4888–901. doi: 10.1245/s10434-023-13498-0 (PMC10319676; doi:10.1245/s10434-023-13498-0)
Supplement: Supplementary file 1 — Supplementary file1 (DOCX 4465 kb) [file 10434_2023_13498_MOESM1_ESM.docx]

**Supplementary Online Material**

**eTable 1.** Studies excluded from the systematic review.

**eTable 2.** Newcastle-Ottawa score for the included studies.

**eFigure 1.** Forest plot of surgery duration meta-analysis.

**eFigure 2.** Forest plot of blood loss meta-analysis.

**eFigure 3.** Forest plot of intraoperative transfusion meta-analysis.

**eFigure 4.** Forest plot of hospital stay meta-analysis.

**eFigure 5.** Forest plot of perioperative mortality meta-analysis.

**eFigure 6.** Forest plot of overall morbidity meta-analysis.

**eFigure 7.** Forest plot of major complications meta-analysis.

**eFigure 8.** Forest plot of R0 resection meta-analysis.

**eFigure 9.**

- **eFigure 9A.** Forest plot of number of lymphadenectomy performance.
- **eFigure 9A.** Forest plot of number of lymph nodes retrieved meta-analysis.

**eFigure 10.** Forest plot of “two stage” hazard of death meta-analysis.

**1. STUDY SELECTION**

| **eTable 1.** Studies excluded from the systematic review | | |
| --- | --- | --- |
| **Study** | **PMID** | **Cause of exclusion** |
| Postoperative survival of extrahepatic and intrahepatic cholangiocarcinoma after surgery: a population-based cohort | **35414539** | Include extrahepatic ICC |
| Propensity score matched comparison of robotic and open major hepatectomy for malignant liver tumors | **34981238** | Include other type of tumors |
| Selection criteria for minimally invasive resection of intrahepatic cholangiocarcinoma—a word of caution: a propensity score matched analysis using the national cancer database | **34750709** | Include robotic surgery |
| Safety and feasibility of laparoscopic versus open liver resection with associated lymphadenectomy for intrahepatic cholangiocarcinoma | **32921695** | No PSM or RCT study |
| Comparison of perioperative and oncologic outcomes between open and laparoscopic liver resection for intrahepatic cholangiocarcinoma | **26902611** | No PSM or RCT study |
| Laparoscopic liver resection for intrahepatic cholangiocarcinoma: a single-center experience | **32503376** | No PSM or RCT study |
| Indications for laparoscopic liver resection of mass-forming intrahepatic cholangiocarcinoma | **0924307** | No PSM or RCT study |
| Laparoscopic approach to intrahepatic cholangiocarcinoma is associated with an exacerbation of inadequate nodal staging | **30895496** | No PSM or RCT study |
| Intrahepatic cholangiocarcinoma as the new field of implementation of laparoscopic liver resection programs. A comparative propensity score-based analysis of open and laparoscopic liver resections | **32342213** | Duplicated data |
| Can laparoscopic liver resection provide a favorable option for patients with large or multiple intrahepatic cholangiocarcinomas? | **28032221** | No PSM or RCT study |
| Laparoscopic liver resection for intrahepatic cholangiocarcinoma | **25789408** | No PSM or RCT study |
| Safety and feasibility of laparoscopic liver resection with associated lymphadenectomy for intrahepatic cholangiocarcinoma: a propensity score-based case-matched analysis from a single institution | **26194257** | Duplicated data |
| The role of minimally invasive surgery in the treatment of cholangiocarcinoma | **28292628** | Review |
| Advanced laparoscopic HPB surgery: Experience in Seoul National University Bundang Hospital | **32490336** | Review |
| Total laparoscopic vs. open liver resection: comparative study with propensity score matching analysis | **32428137** | Insufficient data |

**2. ASSESSMENT OF METHODOLOGICAL QUALITY**

| **eTable 2.** Newcastle-Ottawa score for the included studies | | | | | | | | | |
| --- | --- | --- | --- | --- | --- | --- | --- | --- | --- |
| **First author, year** | Representativeness of cohort | Selection of non-exposed cohort | Ascertainment of exposure | Demonstration that outcome of interest was not present at start of study | Comparability of cohorts on the basis of the design or analysis | Assessment of outcome | Was follow-up long enough for outcomes to occur | Adequacy of follow up of cohorts | Total score |
| **Jinhuan et al (2022)** | ★ | ★ | ★ | ★ | ★ | ★ | ★ | ★ | **8** |
| **Ratti et al (2021)** | ★ | ★ | ★ | ★ | ★ | ★ | ★ | ★ | **8** |
| **Brustia et al (2021)** | ★ | ★ | ★ | ★ | ★ | ★ | ★ | ★ | **8** |
| **Hobeika et al (2021)** | ★ | ★ | ★ | ★ | **-** | ★ | ★ | ★ | **7** |
| **Kang et al (2020)** | ★ | ★ | ★ | ★ | - | ★ | ★ | **-** | **6** |
| **Zhu et al (2019)** | ★ | ★ | ★ | ★ | **-** | ★ | ★ | ★ | **7** |

**3. AGGREGATED DATA META-ANALYSIS**

*3.1 OPERATIVE TIME*

Data of Egger’s test

**
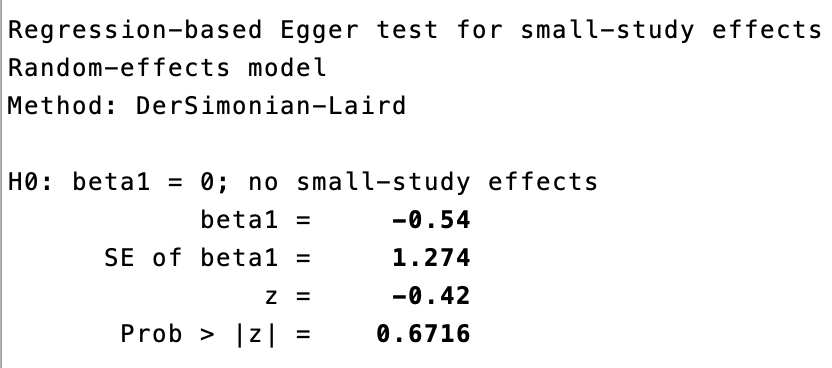
**

Funnel plot showing publication bias; Mean difference operative time (minutes) (X-axis) with it is standard error (Y-axis)

**eFigure 1.** Forest plot of surgery duration meta-analysis.

*3.2 BLOOD LOSS*

Data of Egger’s test


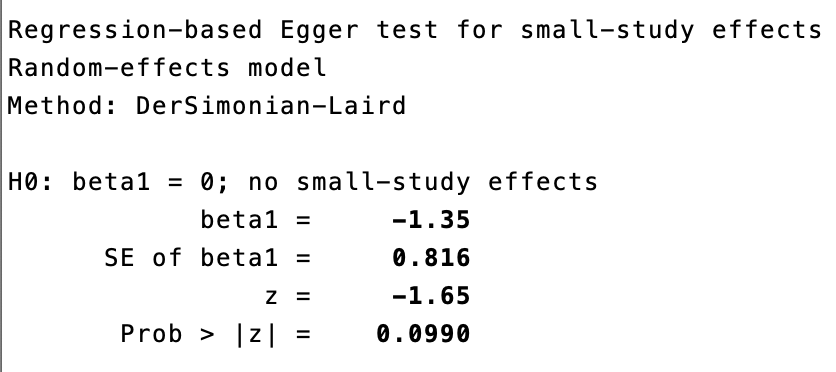


Funnel plot showing publication bias; Mean difference of blood loss (ml) (X-axis) with it is standard error (Y-axis)

**eFigure 2.** Forest plot of blood loss meta-analysis.

*3.3 INTRAOPERATIVE TRANSFUSION*

Data of Egger’s test

**
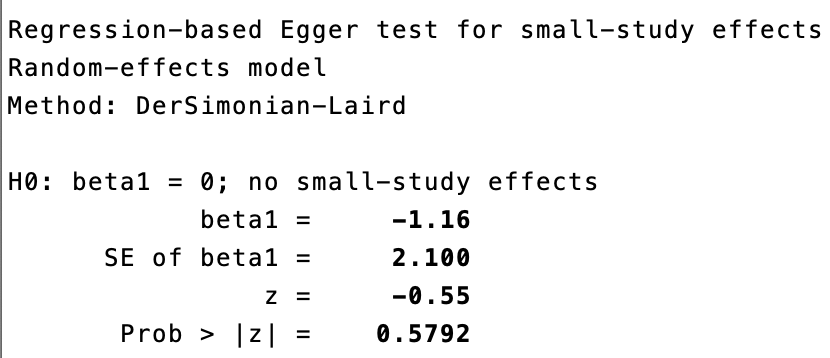
**

Funnel plot showing publication bias; intraoperative transfusion (log OR) (X-axis) with it is standard error (Y-axis)

**eFigure 3.** Forest plot of intraoperative transfusion meta-analysis.

*3.4 LENGTH OF HOSPITAL STAY*

Data of Egger’s test


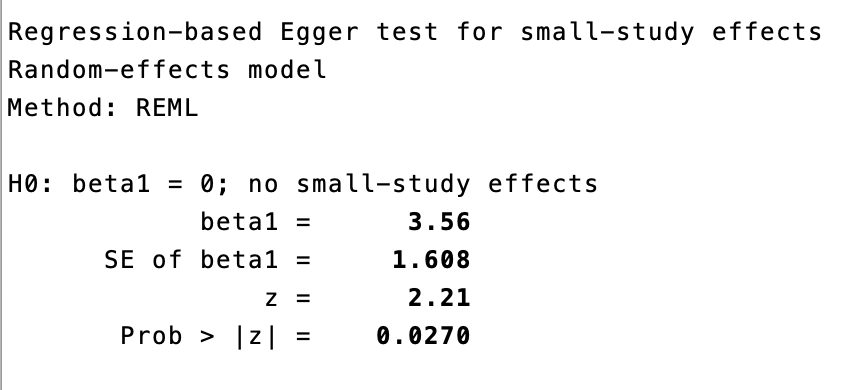


Funnel plot showing publication bias; Mean difference of hospital stay (days) (X-axis) with it is standard error (Y-axis)

**eFigure 4.** Forest plot of hospital stay meta-analysis.

*3.5 PERIOPERATIVE MORTALITY*

Data of Egger’s test


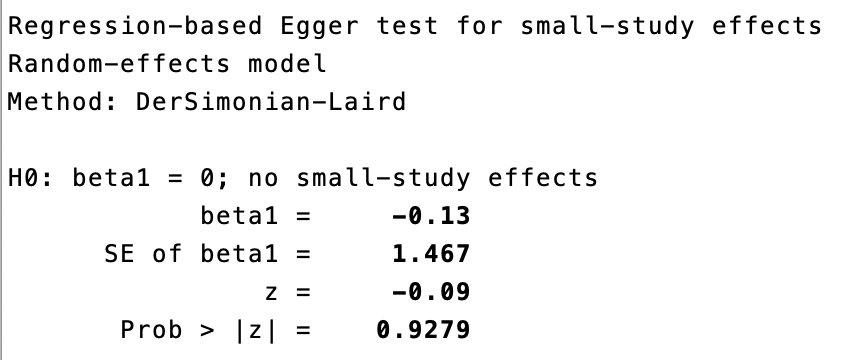


Funnel plot showing publication bias; perioperative mortality (log OR) (X-axis) with it is standard error (Y-axis)

**eFigure 5.** Forest plot of perioperative mortality meta-analysis.

*3.6 OVERALL MORBIDITY*

Data of Egger’s test


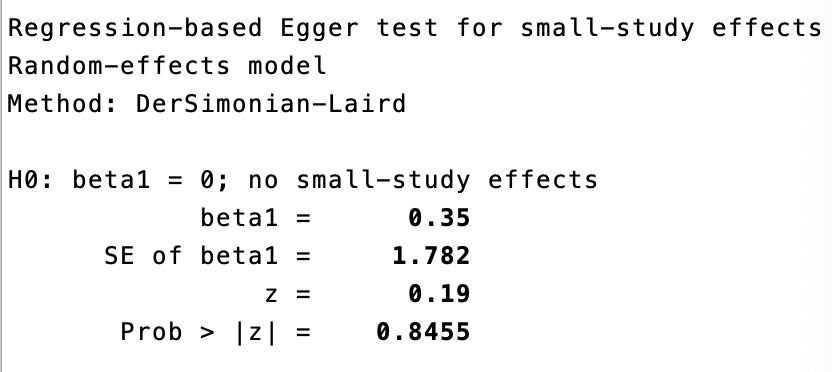


Funnel plot showing publication bias; overall morbidity (log OR) (X-axis) with it is standard error (Y-axis)

**eFigure 6.** Forest plot of overall morbidity meta-analysis.

*3.7 MAJOR COMPLICATIONS (CLAVIEN-DINDO≥ 3)*

Data of Egger’s test

**
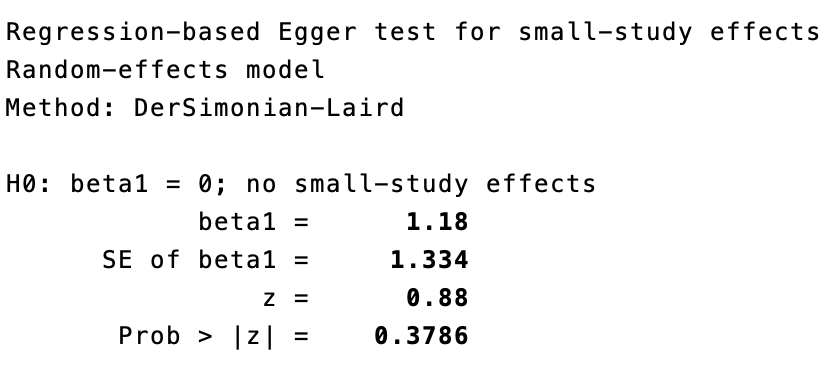
**

Funnel plot showing publication bias; major complications (logOR) (X-axis) with it is standard error (Y-axis)

**eFigure 7.** Forest plot of major complications metanalysis.

*3.8 R0 RESECTION*

Data of Egger’s test


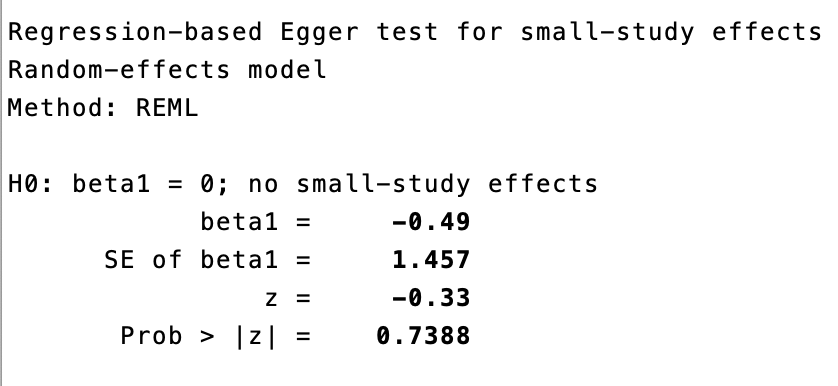


Funnel plot showing publication bias; risk of R0 resection (logOR) with it is standard error (Y-axis)

**eFigure 8.** Forest plot of R0 resection meta-analysis.

*3.9 LYMPHADENECTOMY AND LYMPH NODE RETRIEVAL*

**
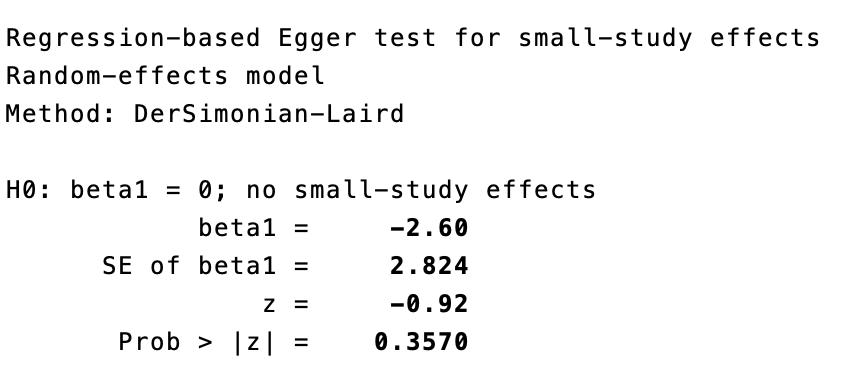
**Data of Egger’s test

Funnel plot showing publication bias; risk of lymphadenectomy (logOR) with it is standard error (Y-axis)

**eFigure 9A.** Forest plot of number of lymphadenectomy performance.

**eFigure 9B.** Forest plot of number of lymph nodes retrieved meta-analysis.

*3.10 HAZARD OF DEATH (“TWO STAGE META-ANALYSIS”)*

Data of Egger’s test


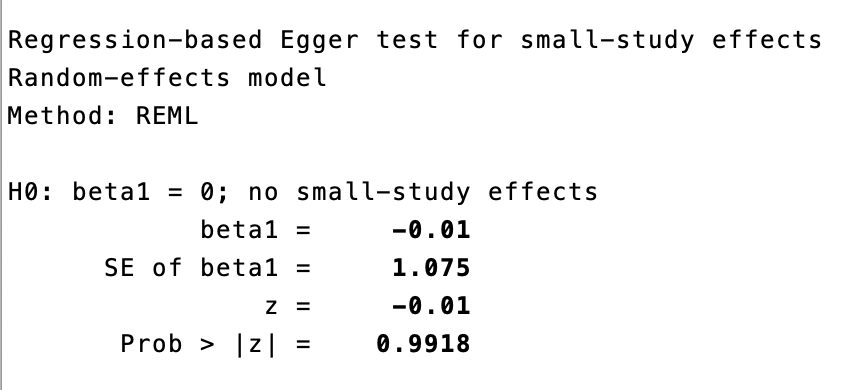


Funnel plot showing publication bias; risk of R0 resection (logHR) with it is standard error (Y-axis)

**eFigure 10.** Forest plot of “two stage” hazard of death meta-analysis

**4. RECONSTRUCTION OF INDIVIDUAL PATIENT DATA SURVIVAL**

**Jinhuan et al. (2022)**

Kaplan Meier curve from original paper


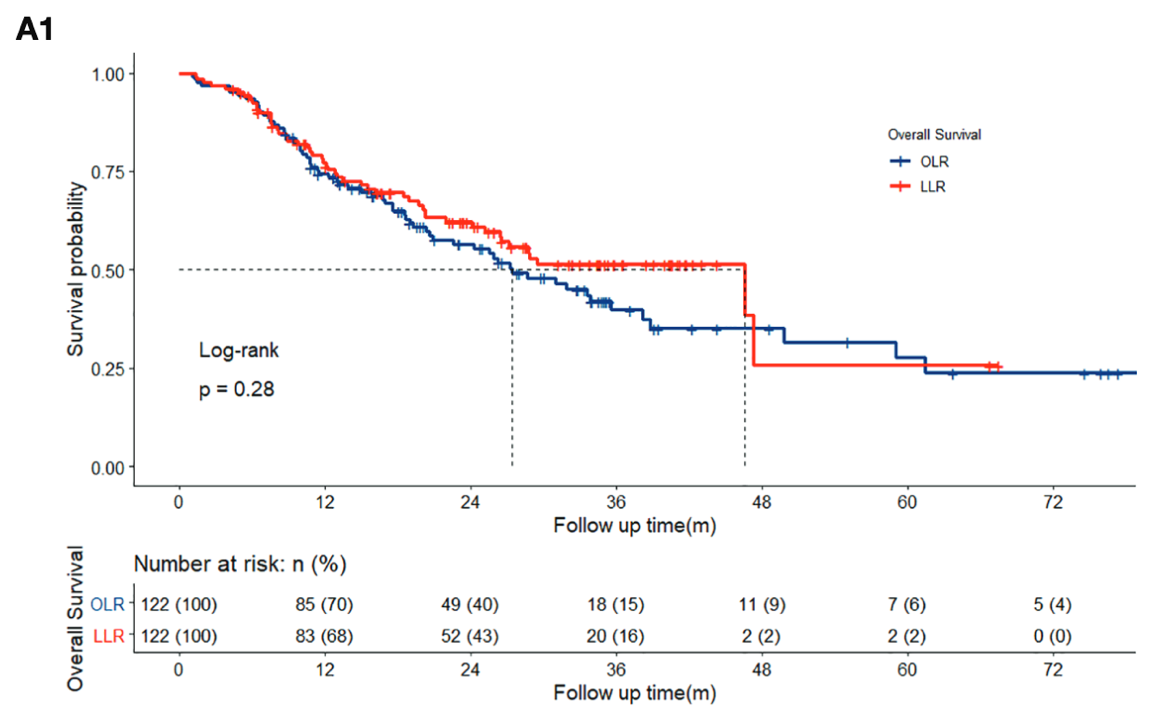


Reconstructed survival curves - including number-at-risk tables-.

Grambsch-Therneau test


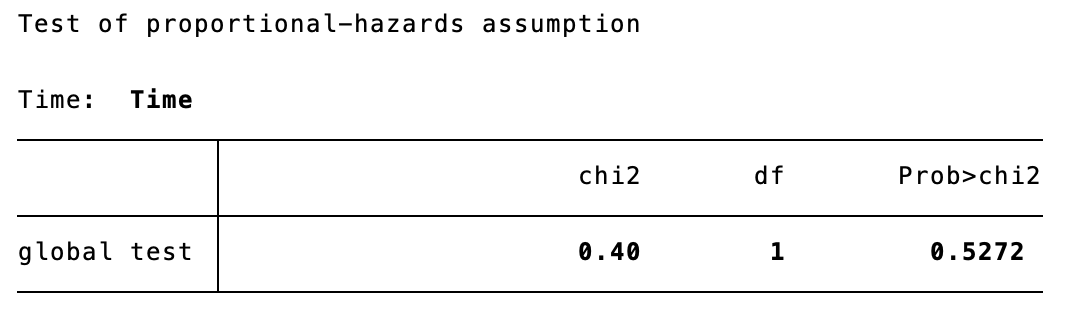


Schoenfeld residuals plot

Predicted versus observed survivor functions

**Ratti et al (2021)**

Kaplan Meier curve from original paper


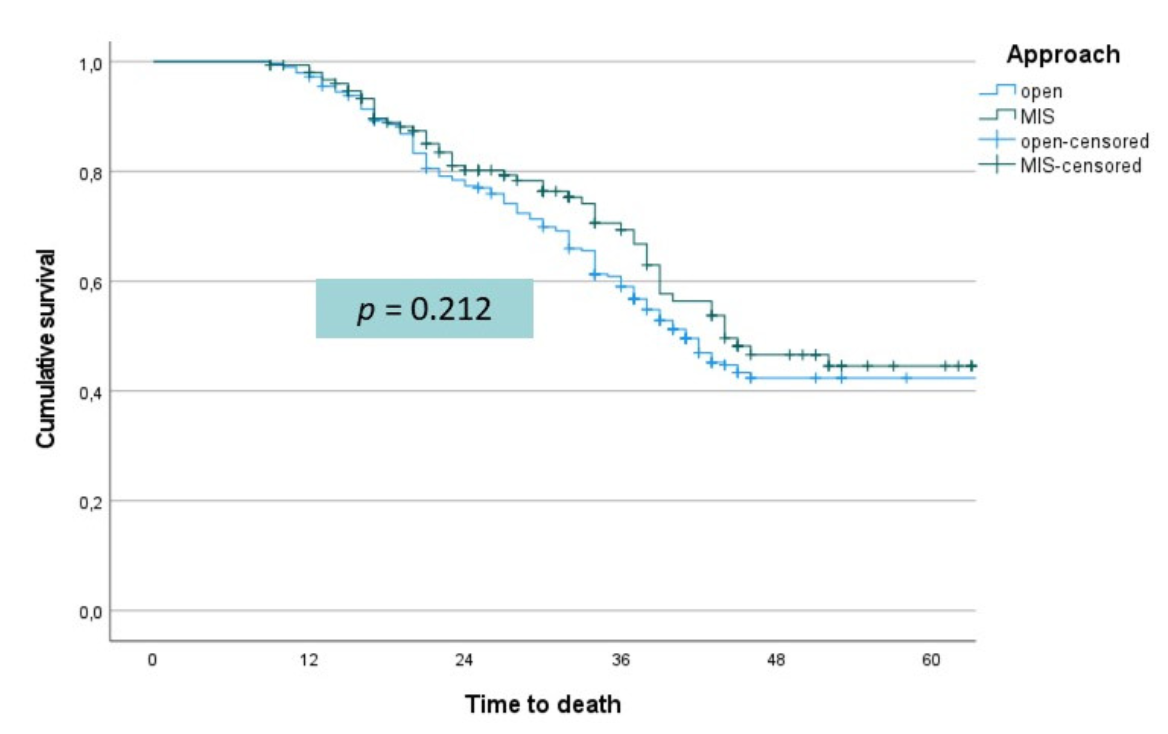


Reconstructed survival curves - including number-at-risk tables

Log rank test


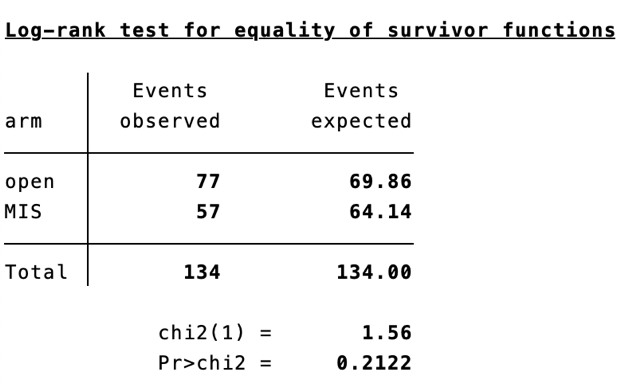


Grambsch-Therneau test


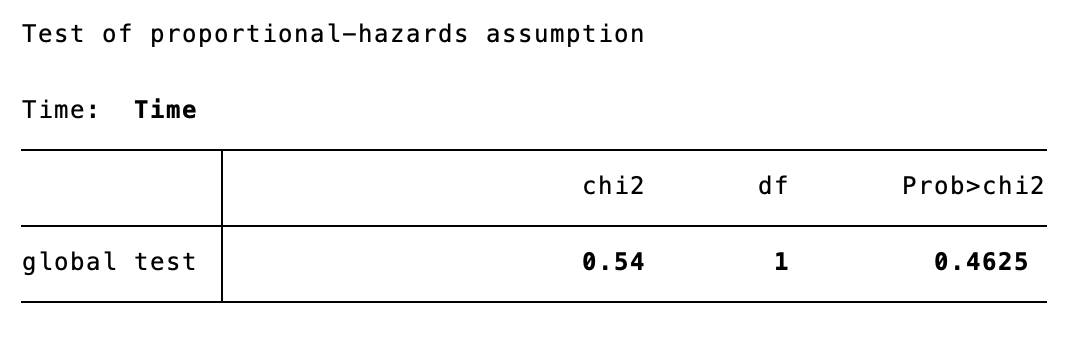


Schoenfeld residuals plot

Predicted versus observed survivor functions

**Brustia et al (2021)**

Kaplan Meier curve from original paper


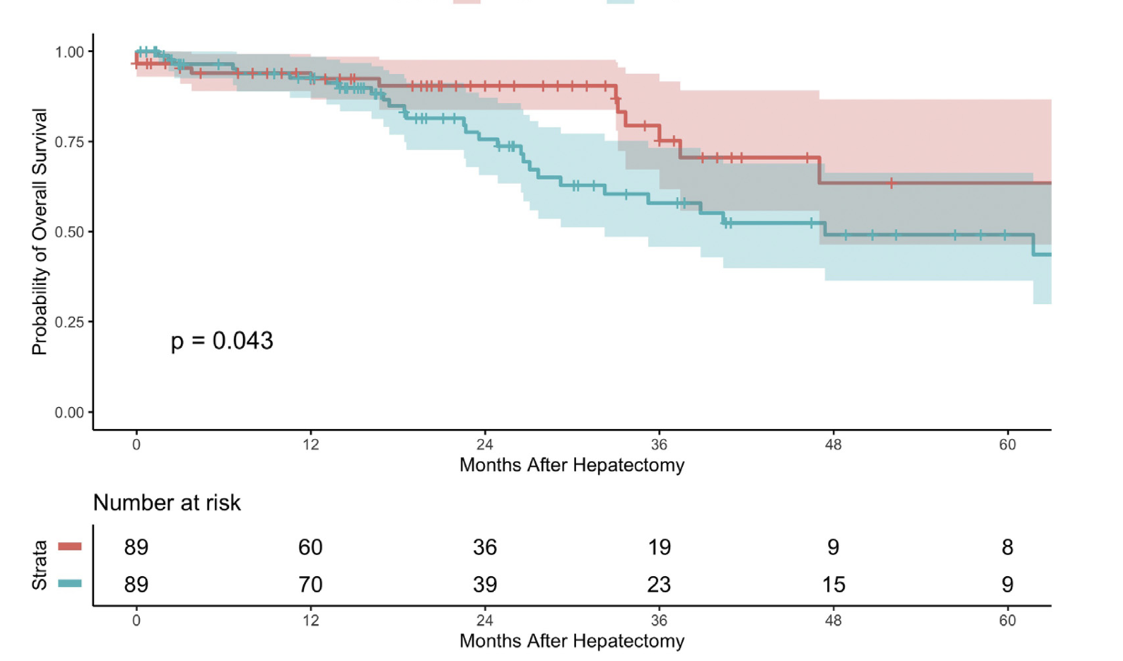


Reconstructed survival curves - including number-at-risk tables-

Grambsch-Therneau test


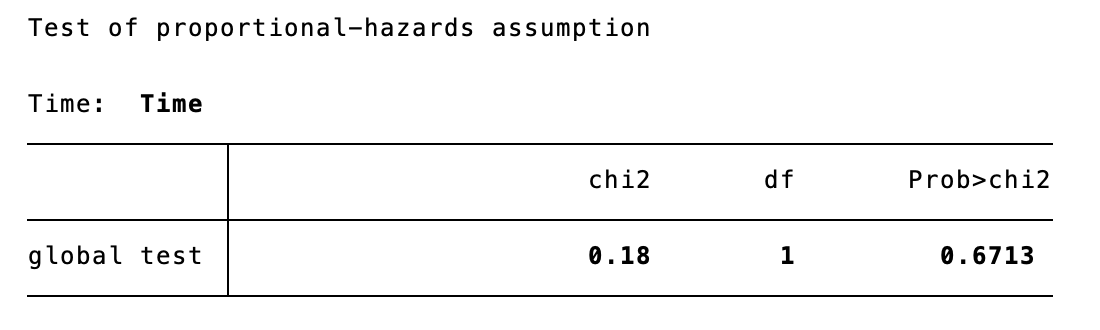


Schoenfeld residuals plot

Predicted versus observed survivor functions

**Kang et al (2020)**

Kaplan Meier curve from original paper


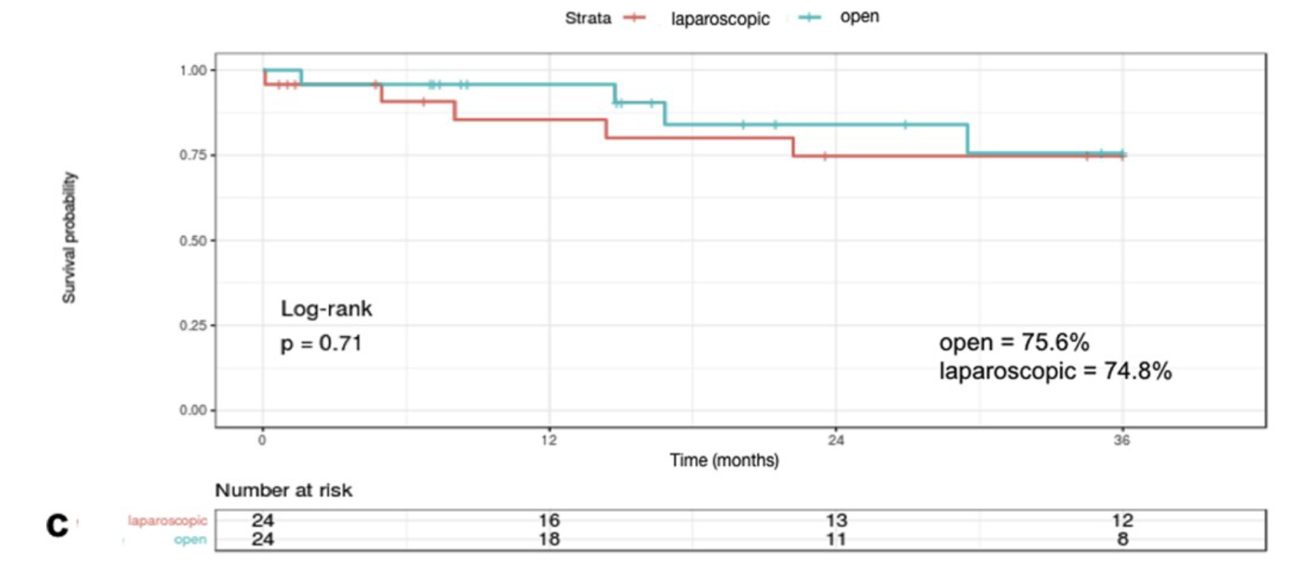


Reconstructed survival curves - including number-at-risk tables-.

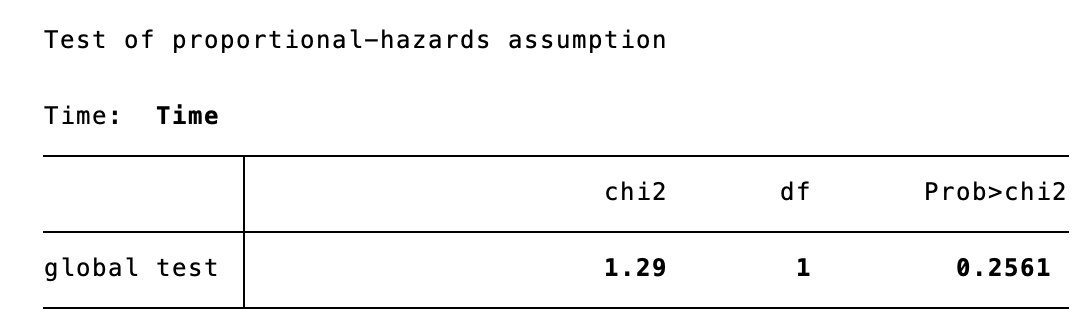
Grambsch-Therneau test

Schoenfeld residuals plot

Predicted versus observed survivor functions

**Zhu et al (2019)**

Kaplan Meier curve from original paper


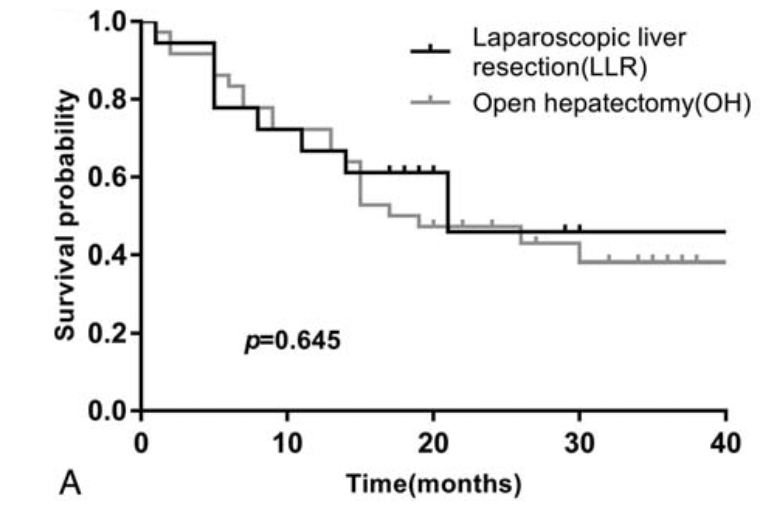


Reconstructed survival curves - including number-at-risk tables-.

Log rank test


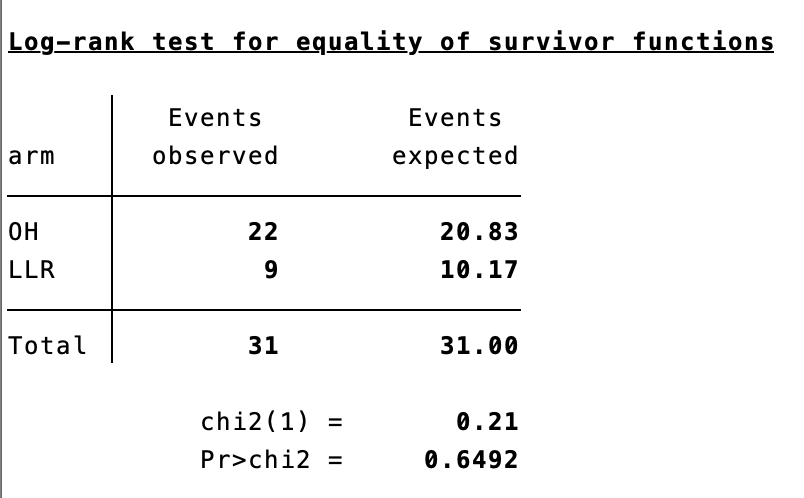


Grambsch-Therneau test


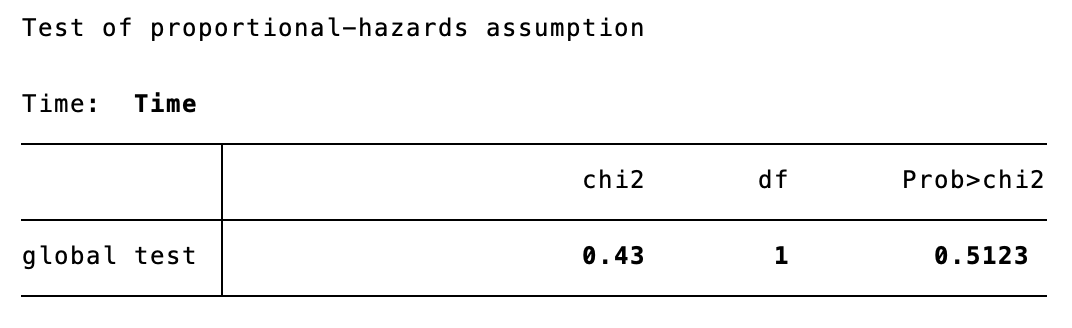


Schoenfeld residuals plot

Predicted versus observed survivor functions

**Pooled cohort**

Reconstructed survival curves - including number-at-risk tables-.


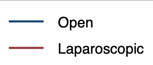


Grambsch-Therneau test


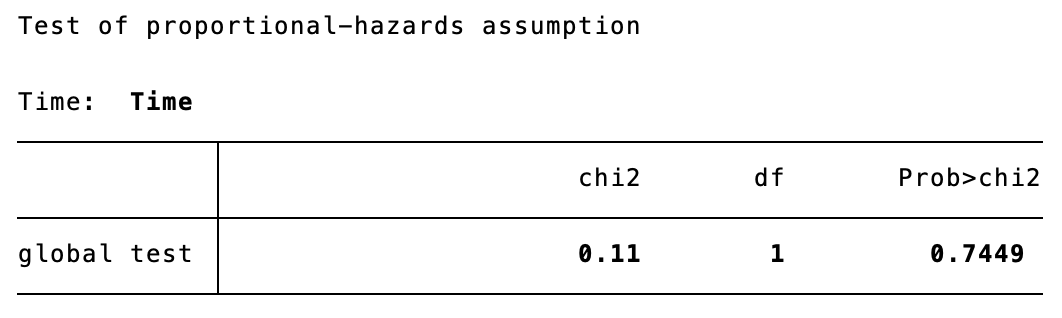


Schoenfeld residuals plot

Predicted versus observed survivor functions
